# Chloride Ions: Essential Agents for Achieving Spontaneous Delamination of MXene

**DOI:** 10.1002/adma.74422

**Published:** 2026-08-19

**Authors:** Sukhyeun Jang, Yong‐Jae Kim, Incheol Jeong, Minju Kim, Jaewoong Lee, Jihan Kim, Ki‐Min Roh, Kang Taek Lee, Seon Joon Kim, Yonghee Lee, Chi Won Ahn, Hee‐Tae Jung

**Affiliations:** ^1^ Department of Chemical and Biomolecular Engineering Korea Advanced Institute of Science and Technology (KAIST) Daejeon Republic of Korea; ^2^ National Nanofab Center Korea Advanced Institute of Science and Technology (KAIST) Daejeon Republic of Korea; ^3^ Resources Utilization Research Center Korea Institute of Geoscience and Mineral Resources Daejeon Republic of Korea; ^4^ Department of Mechanical Engineering Korea Advanced Institute of Science and Technology Daejeon Republic of Korea; ^5^ Convergence Research Center for Solutions to Electromagnetic Interference for Future‐mobility and Extreme Materials Research Center Korea Institute of Science and Technology Seongbuk‐gu Seoul Republic of Korea; ^6^ Nano & Information Technology, KIST School University of Science and Technology Seongbuk‐gu Seoul Republic of Korea; ^7^ Department of Nano & Advanced Materials Science and Engineering Kyungpook National University Sangju‐si Gyeongsangbuk‐do Republic of Korea

**Keywords:** delamination, etching, intercalation, MXenes, synthesis mechanism, transparent conductive films

## Abstract

Despite significant advancements in MXene synthesis, the etching and delamination processes remained largely empirical, with limited understanding of their underlying mechanisms. Here, we uncover the pivotal role of chloride ions (Cl^–^) in enabling spontaneous delamination by facilitating Li^+^ ion intercalation through interlayer expansion and surface termination during the etching. In all cases, delamination is not observed where Cl^−^ was absent in the etchant, and spontaneous delamination occurs only in samples where chlorine terminations are formed during the etching. Our findings are utilized to develop a one‐step, maximized spontaneous MXene synthesis method with high delamination efficiency, yielding an 83.6% production rate and an enhanced electrical conductivity of ∼15 000 S/cm. It shows superior performance in transparent conductive films and Joule heating devices, showcasing their potential for next‐generation electronic and energy systems. This delamination mechanism and synthesis approach are not limited to Ti_3_AlC_2_ MAX but can be extended to other MAX phases, highlighting their broad applicability. Observations made in this effort should serve as the foundation for designing new synthesis routes for diverse MAX phases and advancing MXene‐based applications.

## Graphical abstract

This work first identifies chloride ions as essential agents for spontaneous MXene delamination. Without Cl^−^, etched Ti_3_C_2_T_x_ remains multilayered; with Cl^−^, interlayer modification enables Li^+^ intercalation and osmotic swelling. The SPEED process couples HF‐assisted Al extraction, Cl^−^‐mediated interlayer expansion, and Li^+^‐driven swelling to produce high‐yield, conductive, oxidation‐resistant MXenes.

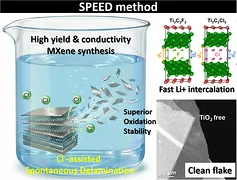

## 1 Introduction

MXenes, a novel class of two‐dimensional (2D) transition metal carbides, nitrides, and carbonitrides, were first discovered in 2011 [1] and have since emerged as a significant addition to the 2D material family. Due to their exceptional electrical conductivity and dense distribution of hydrophilic functional groups, MXenes have been widely explored for applications in electronic devices [2, 3, 4], energy storage [5, 6, 7, 8, 9, 10], sensors [11, 12, 13], electromagnetic shielding [14, 15, 16], and optoelectronic/photonic devices [17, 18, 19, 20, 21]. MXenes are synthesized from MAX phase precursors with the general formula M_n+1_AX_n_, where M is a transition metal, A is typically a group 13 or 14 element, X is carbon and/or nitrogen, and *n* ranges from 1 to 4 [22, 23]. Their synthesis involves two key steps: (1) selective etching of the A layer from the ceramic MAX phase and (2) delamination of the resulting MXene sheets, where intercalants facilitate layer separation [24, 25, 26, 27]. The synthesis conditions, such as etchant concentration, reaction temperature, and processing time, significantly influence the final properties of MXenes [28, 29, 30, 31, 32, 33].

In the etching process, various wet etchants have been employed to remove M–A bonds. Early methods utilized hydrofluoric acid (HF) directly [1], while more recent approaches involve in‐situ HF generation using lithium fluoride (LiF) and hydrochloric acid (HCl) to selectively remove aluminum layers [23]. Additionally, mixed HF/HCl solutions have been introduced to enhance electrical properties and production yield [34]. Beyond liquid‐phase etching, molten salt‐based methods have also been explored, offering an alternative pathway for MXene synthesis [35, 36]. Following etching, delamination is required to separate the stacked MXene layers into single sheets. Lithium‐based intercalants such as LiCl and LiF are commonly used [37, 38], as the small Li^+^ ions electrostatically interact with negatively charged surface groups, leading to an expanded interlayer structure and reduced van der Waals forces for easier exfoliation [38, 39, 40, 41, 42]. Additionally, organic intercalants such as dimethyl sulfoxide (DMSO), tetrabutylammonium hydroxide (TBAOH), and tetramethylammonium hydroxide (TMAOH) have been widely utilized for delamination [24, 25, 37, 43].

Since MXene etching and delamination occur at the atomic scale [44], the process is highly sensitive to reaction conditions. Optimized synthesis methods are crucial to ensuring high efficiency, structural integrity, and minimal material degradation. In particular, spontaneous delamination is highly desirable, as it maximizes yield while preserving intrinsic material properties [23, 38, 45, 46]. This enables MXenes to be suitable for various advanced applications while maintaining its structural integrity without additional processing techniques. However, while extensive research has focused on post‐synthesis treatments of MXenes for various applications, relatively little attention has been given to the fundamental mechanisms governing the initial etching and delamination processes. Thus, it is important to understand the interactions between different etching and delamination chemistries at the atomic level and the specific roles of individual ions involved, in order to achieve innovative progress in MXene synthesis.

In this study, we demonstrate for the first time that Cl^−^ ions play a pivotal role in enabling efficient ion intercalation and spontaneous MXene delamination. Our experimental findings reveal that delamination occurs only when Cl^−^ is present in the etching solution, which acts as a prerequisite for the effective intercalation of Li^+^ ions, thereby enabling spontaneous delamination that cannot be achieved by Li^+^ ions alone in the absence of Cl^−^. Computational calculations further show that Cl^−^ ions attached to the MXene surface increase interlayer spacing, thereby enhancing Li^+^ intercalation and facilitating effective delamination. Based on these insights, we developed an optimized synthesis method that integrates etching and delamination, achieving high‐yield and high‐quality MXene production. This Cl^−^‐assisted approach reduces defect formation during the etching and improves oxidation stability, expanding the potential of MXenes for industrial applications.

## 2 Synthetic Mechanism

### 2.1 Critical Role of Cl^−^ Ions in Facilitating MXene Delamination During Synthesis

To reveal the mechanism of spontaneous delamination, we systematically designed the etching process by varying the presence of two key ions: chlorine ions (Cl^−^) and lithium ions (Li^+^). HF served as the base etchant, with different solutions providing Cl^−^ and Li^+^ ions either separately or together. The etching conditions were classified as follows: HF only (control), HF/LiF (Li^+^ only), HF/HCl (Cl^−^ only), HF/LiCl (both ions present), and HF/HCl/LiCl (both ions combined). Additionally, the conventional spontaneous delamination method using LiF/HCl (in‐situ HF etching with both ions) was evaluated.

Among the various conditions, spontaneous delamination occurred in certain cases during the washing process. As shown in Figure 1, etching solutions without Cl^−^ ions, such as HF or HF/LiF (route 1: without Cl^−^), did not result in delamination, leaving the MXene multilayered even after subsequent Li^+^ intercalation with LiCl. In contrast, etching solutions containing only Cl^−^ ions (HF/HCl) enabled delamination after secondary addition of Li^+^ ions during the delamination step (upper section of route 2 in Figure 1). Notably, etching solution containing both Cl^−^ and Li^+^ ions (HF/LiCl, HF/HCl/LiCl, and LiF/HCl) facilitated spontaneous delamination (bottom section of route 2 in Figure 1), yielding a significant amount of few‐layer MXene in the supernatant. Our systematic investigation demonstrates that the presence of Cl^−^ ions in the etching solution is a fundamental prerequisite for expanding the interlayer spacing of multilayer MXene. This initial expansion lowers the energetic barrier for subsequent Li^+^ intercalation which then acts as the primary driver for the osmotic swelling required for spontaneous MXene delamination. We hypothesize that chlorine termination occurs during the etching process, enhancing Li^+^ intercalation when Cl^−^ ions are present in the initial etching solution (Figure 1, route 2). These findings are further supported by extensive experimental results summarized in Table S1.

**FIGURE 1:**
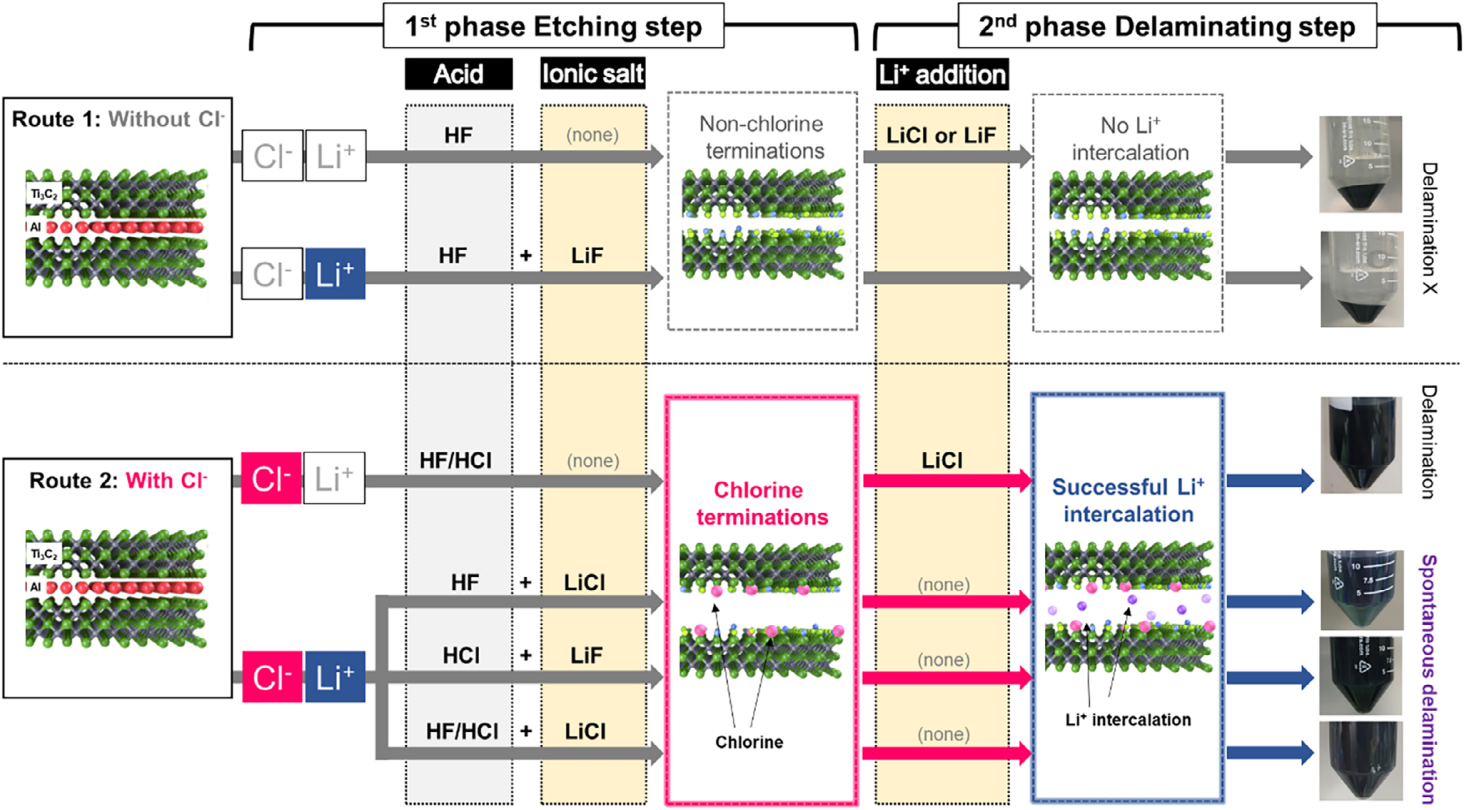
Schematic illustration of systematically designed etching & delamination processes depending on etching solution contents. Route 1 (without Cl^−^ ion) shows only sediment (multi‐layer MXene) after the synthesis due to no delamination. Route 2 (with Cl^−^ ion) occurs delamination after synthesis with chlorine terminations and successful Li^+^ intercalation, in particular, co‐existence of Cl^−^ and Li^+^ ions induced spontaneous delamination (bottom section of route 2).

To investigate the influence of Cl^−^ ions and validate the delamination mechanism, we analyzed the structural and morphological differences arising from various etching solution compositions using XRD, SEM, and TEM. As illustrated in Figure 2, distinct differences in crystalline and microscopic morphology were observed depending on the etching solution composition. The XRD pattern (Figure 2a–c) shows that the (002) peak of MAX phase, located around 9.5° (*d*‐spacing: 0.93 nm) [44], shifted leftward after etching depending on the solution composition. After etching with HF solution, the (002) peak corresponding to multi‐layer MXene appeared slightly left‐shifted and centered at 9.05° (*d*‐spacing: 0.98 nm) [44], and exhibited a broad shape. In the HF/HCl solution, the peak shifted further to 8.21° (*d*‐spacing: 1.08 nm) [44], and we can expect that interlayer spacing is expanded by Cl^−^ ions influence. Notably, after the HF/LiCl solution, a strong peak emerged at 7.18° (*d*‐spacing: 1.23 nm), indicating swollen multi‐layer MXene due to Li^+^ ions intercalation [42, 47, 48], with further increased *d*‐spacing compared to HF/HCl.

**FIGURE 2:**
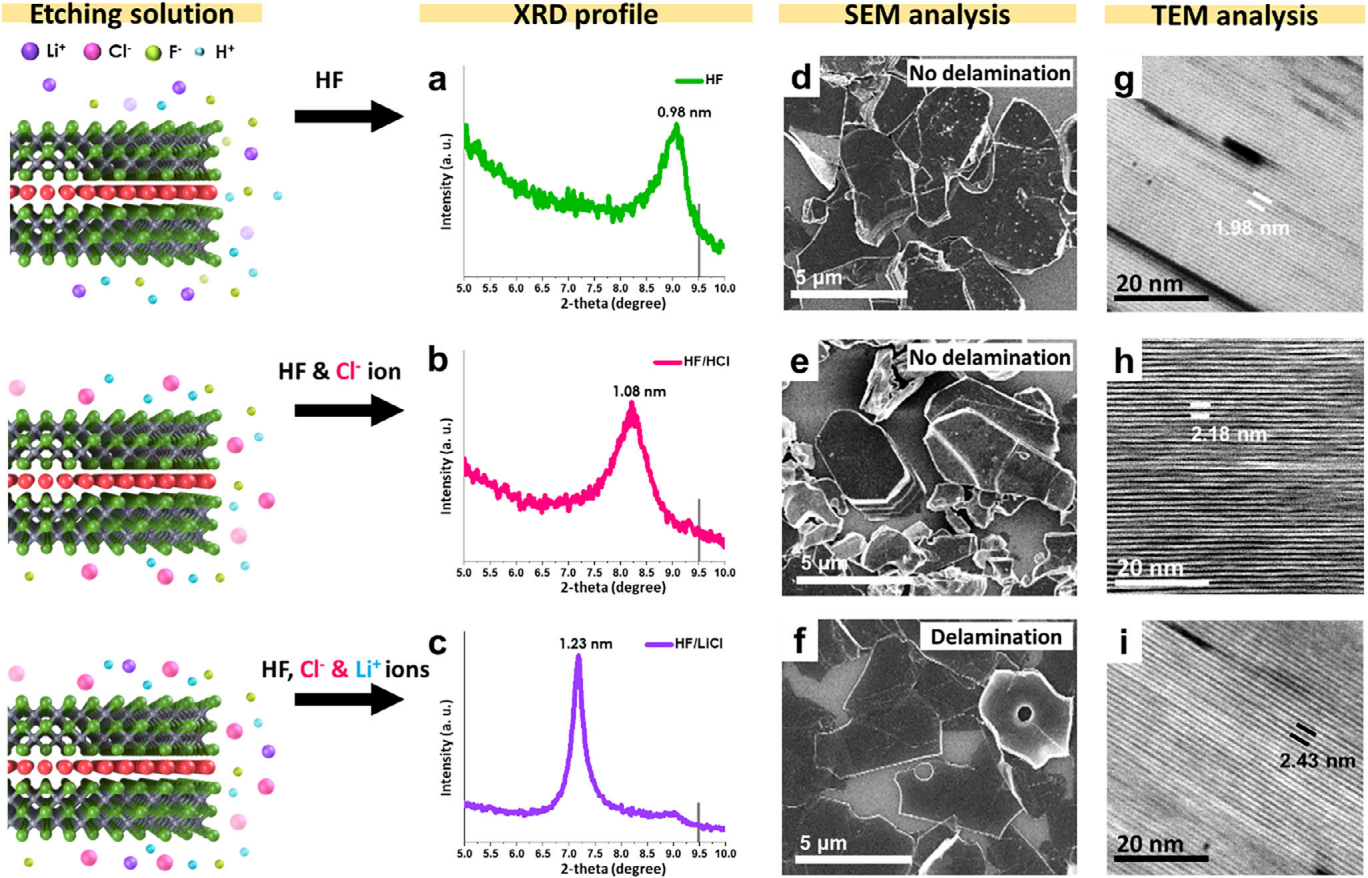
Structural and morphological changes in MXene depending on etching solution: Three kinds of etching solutions (HF, HF/HCl, and HF/LiCl) on the left side were used for XRD, SEM, and TEM analysis. XRD profiles, SEM, and TEM images were obtained from the prepared samples after (a), (d), and (g), HF etching, (b), (e), and (h), HF/HCl etching and (c), (f), and (i), HF/LiCl etching. (Gray lines in the XRD profile indicate (002) peak of pristine MAX phase).

These results, along with SEM analysis (Figure 2d–f; Figure S1), highlight the importance of both Cl^−^ and Li^+^ ions in facilitating delamination. In etching solutions containing both ions (e.g., LiF/HCl and HF/LiCl), delaminated MXene was observed, though the amount differed. HF and HF/HCl solutions showed no delamination, with only polycrystalline and single‐crystalline unetched MAX phase present (Figure 2d,e). In contrast, HF/LiCl produced a relatively large amount of delaminated MXene (Figure 2f). These results suggest that both Cl^−^ and Li^+^ ions are essential for achieving effective interlayer expansion and promoting delamination. However, the exact mechanism by which Cl^−^ ions contribute to delamination has not yet been fully elucidated.

TEM analysis shows that interlayer spacing increased with the addition of Cl^−^ and Li^+^ ions, consistent with the XRD results. The statistical reliability of these interlayer‐spacing changes was further confirmed by five independent XRD measurements from distinct synthesis batches and additional cross‐sectional HR‐TEM analysis covering multiple distinct zones across two individual flakes and at least 10 continuous interlayer regions per flake (Figure S2). Etching with HF alone resulted in the smallest *c*‐lattice parameter of 1.98 nm as shown in Figure 2g, while the HF/HCl etchant increases *c*‐lattice parameter to 2.18 nm (Figure 2h). Also, Figure 2i shows the largest *c*‐lattice parameter of 2.43 nm induced by the etching with HF/LiCl solution [42, 44, 47, 48]. This distinct lattice expansion observed prior to complete delamination confirms that Cl^−^ ions actively functionalize the nascent Ti sites at the moving etching frontier. These findings demonstrate that the high local concentration of Cl^−^ enables real‐time surface passivation during the dynamic Al extraction phase, acting as an immediate structural wedge that prevents the restacking of adjacent layers before a full MXene interface is even established.

### 2.2 Mechanism of Ion Interactions in MXene Etching and Delamination via Atomic‐Resolution Direct Visualization and Chemical Analysis

To directly confirm that Cl^−^ ions attach to Ti edges and expand the interlayer spacing, we prepared cross‐sectional TEM samples for observation. We systematically examined four different samples: (1) pristine MAX phase, (2) HF‐etched, (3) HF and Cl^−^ ions etched, (4) HF and Cl^−^, Li^+^ ions etched. At first, atomic‐resolution TEM observation was conducted to directly visualize the structural characteristics of the etched Ti_3_C_2_T*_x_* layers and investigate the interaction mechanism of Cl^−^ ions. Figure 3a–d presents an atomic‐resolution TEM image from MAX phase to delaminated MXene. During the etching process, atomic Al‐layers in Figure 3a were removed by HF (Figure 3b) or HF/HCl (Figure 3c) etching with slightly increased gap between Ti atoms. Finally, Figure 3d shows the delaminated layers due to Li^+^ ion intercalation into inter‐layers, which are atomic Al‐layer removed. In particular, Figure 3a, where the more exact *d*‐spacing of MAX phase is measured to be 0.94 nm, and it is gradually increasing to 0.99 nm for HF etching and 1.07 nm for HF/HCl etching, similar to XRD results in Figure 2. For more exact comparison, we measured the distance between each pair of the outmost Ti atom (*d*_Ti_) depending on etching solutions and it shows that *d*_Ti_‐spacing is related to *d*‐spacing increase of etched Ti_3_C_2_T*_x_* depending on etching solutions. First, *d*_Ti_‐spacing of MAX phase is confirmed to be 0.48 nm, but *d*_Ti_‐spacing slightly increased to 0.527 nm after etching the Al layer with HF (Figure 3b). However, in Figure 3c, the gap further expanded to 0.607 nm due to Cl^−^ ions attachment after etching with HF/HCl presumably. Interestingly, we observed delaminated Ti_3_C_2_T*_x_* layers in Figure 3d, induced by the combined effect of Cl^−^ attachment and Li^+^ intercalation following HF/LiCl etching. While it was challenging to identify the atomic configuration of Cl^−^ ions bonded to Ti, EDS analysis (Figure S3) allowed us to compare regions where Al etching was fully completed, partially progressed, or not yet initiated. The results revealed that F^−^ ions were present alongside Al in partially etched regions, suggesting that F^−^ ions penetrate deeper into the A layer to facilitate Al removal. In contrast, Cl^−^ ions were detected only in fully etched areas after HF/HCl etching, indicating that Cl^−^ ions primarily interact with Ti sites after Al extraction rather than actively participating in Al removal. Based on these findings, we propose that once Al atoms are eliminated, Cl^−^ ions bond with Ti, occupying the newly exposed sites and modifying the interlayer chemistry. This process involves a sequential role‐sharing mechanism: (i) Selective Al layer extraction by F^−^ ($Ti_{3}\mathrm{Al}C_{2}+3\mathrm{HF}\;\to Ti_{3}C_{2}+\mathrm{Al}F_{3}+1.5H_{2})$ and (ii) immediate surface functionalization by Cl^−^ ($Ti_{3}C_{2}+x\mathrm{HCl}+y\mathrm{HF}+\left(z+w\right)H_{2}O\to Ti_{3}C_{2}Cl_{x}F_{y}O_{z}{\left(\mathrm{OH}\right)}_{w}+\frac{x+y+2z+w}{2}H_{2})$. These equations clarify that Cl^−^ ions serve as structural facilitators that expand the interlayer distance rather than participating in the active chemical removal of Al. The Cl^−^ attachment and its bonding nature was confirmed by XPS analysis (Figure 3e) [36]. The results demonstrated that Cl was indeed bonded to the outmost Ti atoms across all etching conditions containing Cl^−^ ions, as anticipated [36, 41].

**FIGURE 3:**
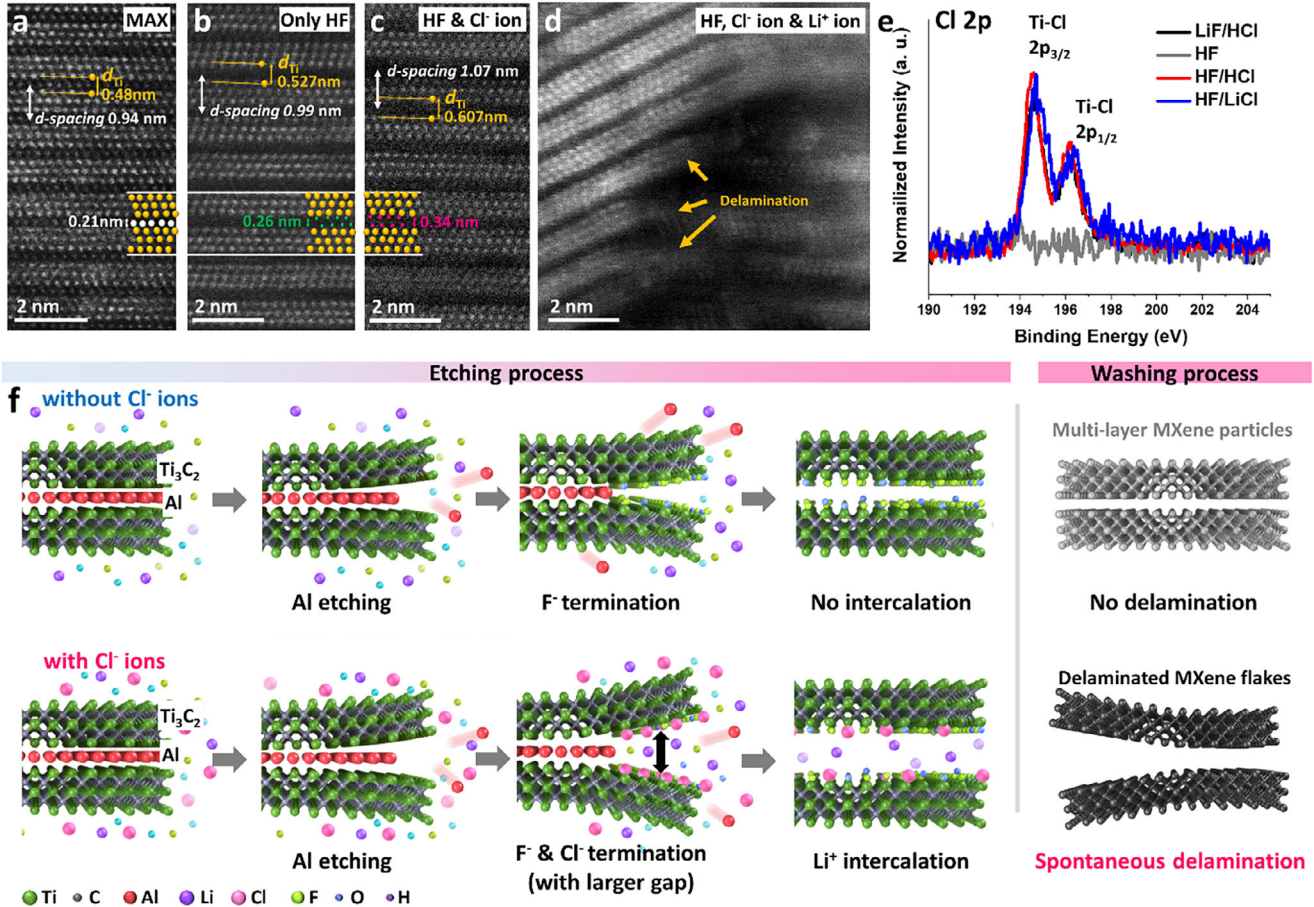
Analysis of structural and compositional changes in Ti_3_C_2_T*_x_* during the etching and delamination. (a–d) Atomic‐resolution TEM images depending on etching solutions: (a) Pristine Ti_3_AlC_2_ MAX, (b) HF‐etched Ti_3_C_2_T_x,_ (c) HF/HCl‐etched Ti_3_C_2_T_x_, and (d) delaminated Ti_3_C_2_T*_x_* layers after HF/LiCl etching. (e) Cl 2p XPS analysis confirming Cl attachment to the outermost Ti atoms. (f) Schematic illustration of the etching and delamination processes without (top) and with (bottom) Cl^−^ ions, highlighting interlayer expansion and Li^+^ intercalation.

From these results, the role of the Cl^−^ ions during the etching and delamination process and mechanism of spontaneous delamination are summarized in Figure 3f. We revealed mechanistic differences in the etching and delamination process with and without Cl^−^ ions. In the absence of Cl^−^ ions (top pathway), Al etching is initiated with HF etching, but the interlayer spacing is not sufficient for effective Li^+^ ion intercalation due to fluorine‐dominant termination, leading to the retention of a multi‐layer structure rather than delamination after washing [39, 40, 44]. In contrast, with Cl^−^ ions (bottom pathway), chlorine termination occurs on the outer Ti_3_C_2_ layers during the etching process, increasing the interlayer spacing and enabling subsequent Li^+^ ions intercalation, which promotes spontaneous delamination during the washing process. This comparison underscores the essential role of Cl^−^ ions in expanding interlayer spacing and promoting efficient delamination during the washing process.

Based on this spontaneous delamination mechanism, we developed a synthetic approach by simultaneously introducing HF, HCl, and LiCl to optimize spontaneous delamination. In this combination, HF serves as the primary etchant, removing Al layers in single‐grain MAX structures and etching grain boundaries in polycrystalline MAX phases with different orientations [44]. Meanwhile, HCl provides Cl^−^ ions, expanding the interlayer spacing between Ti_3_C_2_T*_x_* layers and facilitating effective intercalation of Li^+^ ions from LiCl. Compared to HF alone, the addition of HCl significantly enhances interlayer expansion, likely due to its ability to remove Al atoms more effectively through protonation, which weakens Al–Ti bonding in the MAX phase. Furthermore, Cl^−^ ions intercalate into the layers, increasing the interlayer spacing via electrostatic repulsion and steric hindrance effects [41, 42, 48].

### 2.3 Atomistic Simulations for Understanding the Role of Halogens

To further validate the experimentally proposed mechanism summarized in Figure 3f, density functional theory (DFT) calculations and first‐principles molecular dynamics simulations were performed [49, 50] to elucidate the distinct roles of F^−^ and Cl^−^ ions in the etching and delamination processes. Figure 4 presents the atomistic simulation results based on DFT calculations and first‐principles molecular dynamics to further understand the role of halogens on etching and delamination processes. Figure 4a schematically shows the structure models describing the etching process. It has been suggested that the penetration of solvent via the Ti_3_AlC_2_ MAX edge involves (i) dissociative adsorption of an acid molecule, (ii) accumulation of halogens at the edge, and (iii) extraction of Al [44]. Therefore, we performed DFT calculations to investigate the energy profiles of HF and HCl at each reaction process (Figure 4b). Each reaction energy was obtained by calculating the energy change between the product (Figure 4a) and the reactants (pristine model and acids). Both acids exhibited negative reaction energies for all processes, indicating that the etching process spontaneously occurs. It is interesting to note that the reaction energies for adsorption, accumulation, and extraction were −4.5, −6.6, and −14.3 eV, respectively, for HF, while for HCl, they were −3.7, −6.1, and −10.9 eV. This indicates lower reaction energies throughout the series for HF compared to HCl. The overall reaction involves the binding of halogens on the metals including Ti and Al. Therefore, it is expected that F^−^ ions having higher electronegativity than Cl^−^ ions would predominate in adsorption and halide formation in the initial processes.

**FIGURE 4:**
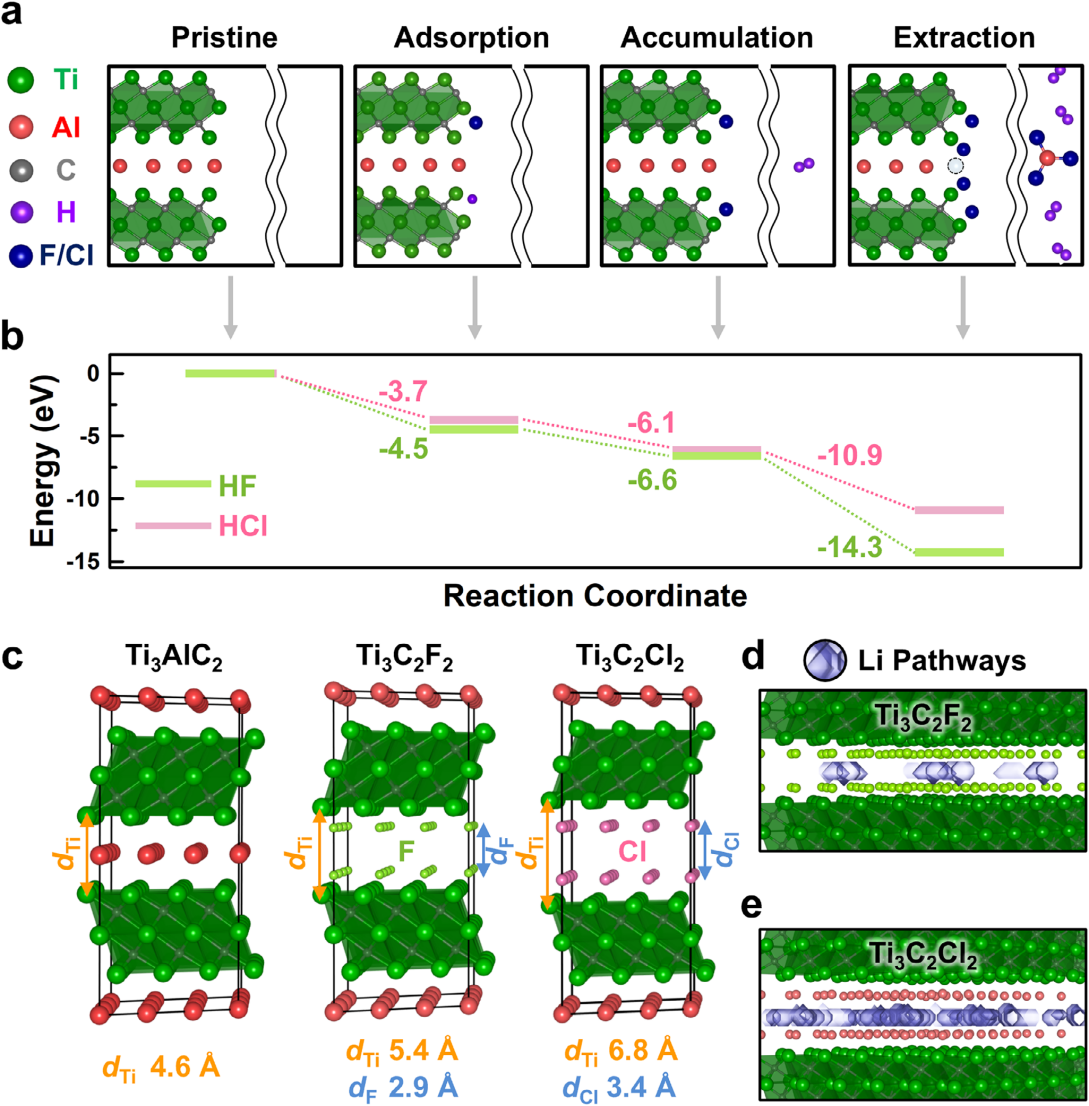
Schematic illustration of etching process and Li^+^ ion diffusion pathways in halogen terminated Ti_3_C_2_ interlayers. (a) Etching process steps: pristine, adsorption, accumulation, and extraction. (b) Reaction energies for HF and HCl. (c) Interlayer spacing for Ti_3_AlC_2_, F^−^‐terminated, and Cl^−^‐terminated structures. Li^+^ ions diffusion pathways in (d) F^−^‐terminated and (e) Cl^−^‐terminated interlayers.

To achieve spontaneous delamination of layers in van der Waals solids, it is necessary to weaken the binding force between layers. In this respect, the role of halogens in the interlayer termination and their subsequent effect on the penetration of Li^+^ ions intercalant should be addressed. Structure models fully terminated by F^−^ ions or Cl^−^ ions were considered to better elucidate the effect of halogens on the interlayer spacing, excluding surface groups such as ─O or ─OH. Figure 4c shows the calculated Ti interlayer spacing (*d*_Ti_) for Ti_3_AlC_2_ MAX (4.6 Å), F^−^‐terminated (5.4 Å), and Cl^−^‐terminated interlayer (6.8 Å). The higher ionic radius of Cl^−^ ion compared to F^−^ ion effectively increased the interlayer spacing. Cl^−^ ions widened *d*_Ti_ by 48% compared to Ti_3_AlC_2_ MAX, while F^−^ ions did 17%. Further, the calculated interlayer spacing between halogen terminations were 2.9 and 3.4 Å for F^−^‒F^−^ (*d*_F_) and Cl^−^‒Cl^−^ (*d*_Cl_), respectively. Therefore, Cl^−^ ions are more likely to provide a significantly larger interlayer spacing rather than participate in Al removal. These results elucidate the observed increase in the interplanar distance in the mixed HF/HCl etching (Figure 3c) compared to single HF etching (Figure 3b) and it strongly supports that Cl^−^ ions facilitate the Li^+^ ion intercalation with a halogen interplanar spacing corresponding to five times the Li^+^ ion radius (∼0.7 Å), while that of F^−^ ions corresponds to four times the Li^+^ ion radius [48]. We also observed that Cl^−^ ions with higher ionic radius would be effective for opening the edge of MAX by causing structural distortions of Ti–Al bond (Figure S5). Figure 4d,e displays the Li^+^ ions diffusion pathways depending on each ion termination. The diffusion pathway of Li^+^ ions is broadly connected in Cl^−^‐terminated environment, whereas it is disconnected and isolated in F^−^‐terminated environment. We believe that Cl^−^ would allow relatively free movement of Li^+^ ions by providing wider interlayer spacing. On the other hand, F^−^ may restrict its mobility due to its narrower interlayer spacing. This phenomenon supports that spontaneous delamination is facilitated by the presence of Cl^−^ ions. To measure the degree of interaction of Li^+^ ions within the interlayer, the binding energy was calculated in Table S2. The binding energies of Li^+^ ion with both terminations were positive, indicating that both halogens would electrostatically attract Li^+^ ion into the interlayer. The interaction of Li^+^ ions with F^−^‐termination (0.69 eV) was stronger than that with Cl^−^‐termination (0.38 eV). These results imply that Cl^−^ would enhance the kinetics by accommodating a large volume while F^−^ would increase the driving force by stronger attraction. Consequently, this mutual complementary cooperation between F^−^ and Cl^−^ would enable a maximized spontaneous delamination of MAX as proposed in Figure 3f.

## 3 Applications

### 3.1 Enhanced Processability and Quality of MXene Synthesized via the SPEED Method

Based on our findings, we suggest the maximized SPEED method (spontaneous process for efficient etching and delamination) to enhance spontaneous delamination while minimizing initial oxidation during synthesis. Notably, using HF/LiCl alone resulted in a yield below 20% (Figure 5a), emphasizing the crucial role of HCl in the etching process. This result suggests that, apart from increasing interlayer spacing, HCl is essential for maintaining an optimal pH environment and facilitating the etching reaction, as observed in the SPEED method. To achieve high etching and delamination efficiency, therefore, we employed HF/HCl/LiCl as the etching solution, carefully optimizing its composition. This approach integrates HF‐based Al etching with Cl^−^‐induced delamination, which significantly improves the overall efficiency. The optimization involved fine‐tuning HF concentrations and reaction times, with detailed conditions and discussions provided in Figures S6–S9.

**FIGURE 5:**
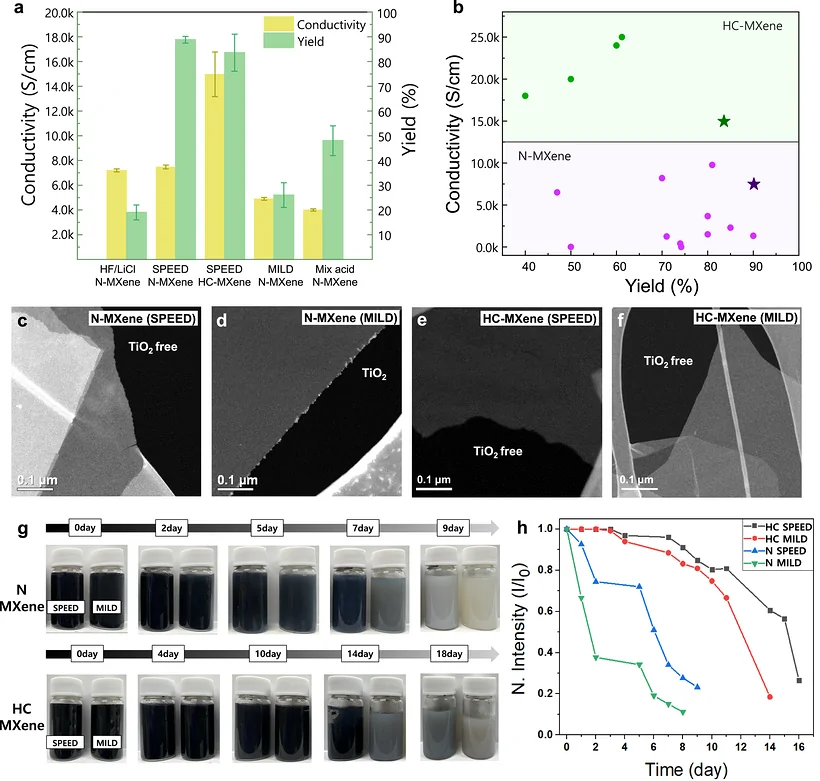
Comparison of production yield, electrical conductivity, and oxidation stability of MXene synthesized by different methods. (a) Yield and electrical conductivity of N‐MXene and HC MXene depending on synthetic methods. (b) State‐of‐the‐art comparison of production yield and electrical conductivity for N‐Ti_3_C_2_T*_x_* MXene and HC‐Ti_3_C_2_T*_x_* MXene (reported literature value; Table S3). (c–f) Initial TEM images of pristine MXene synthesized by in‐house MILD control and SPEED methods. (g) Visual comparison of MXene colloidal solution stability over time at 70°C for SPEED‐MXene and the in‐house MILD‐MXene control. (h) Degradation kinetics of MXene colloidal solutions tracked by normalized intensity (*I*/*I*_0_) at 785 nm.

To sum up, achieving both high production yield and electrical conductivity is best accomplished by conducting the etching reaction at a high HF concentration for a shorter duration and we successfully developed maximized SPEED method and achieved the production of high‐quality normal‐Ti_3_C_2_T*_x_* MXene (N‐MXene) and highly crystalline‐Ti_3_C_2_T*_x_* MXene (HC‐MXene) (Figure 5a). The exceptional electrical conductivity and oxidation stability of HC‐MXene originate from the enhanced structural ordering and the systematic reduction of substitutional oxygen impurities within the internal carbon sublattice during MAX synthesis. In conventional stoichiometric sintering (N‐MAX), volatile Al loss induces localized lattice distortions and carbon vacancies, allowing oxygen infiltration to form a pre‐existing oxycarbide phase [34, 51, 52, 53]. These embedded oxygen atoms persist through the chemical etching process and serve as native sub‐surface defects that act as structural nucleation sites for subsequent TiO_2_ formation [51, 52]. In contrast, synthesizing HC‐MAX with a 1.1–1.2 molar excess of Al compensates for this thermal Al loss, preserving ideal carbon stoichiometry and suppressing initial vacancy formation [34, 51, 53]. By fundamentally eliminating these interior oxygen coordinates, the SPEED‐etched HC‐MXene skips the unstable oxycarbide regime, maintaining a pristine metallic carbide (Ti–C) framework with highly intact layered morphology (Figure S10), which effectively minimizes structural degradation during processing to ensure both high exfoliation yield and efficient charge transport [34, 51, 52, 53]. Unlike the conventional MILD method, in which HF is generated in situ from LiF/HCl, the SPEED process employs a single HF/HCl/LiCl etching solution. In this environment, directly supplied HF promotes efficient Al extraction, while Cl^−^‐assisted interlayer modification and Li^+^‐assisted osmotic swelling occur together during the etching. This single‐pot ion‐coupled process distinguishes SPEED from the LiF/HCl‐based MILD protocol. For the N‐Ti_3_C_2_T*_x_* MXene, well‐modified maximized SPEED method provides high‐quality MXene with ∼7500 S/cm of electrical conductivity and high yield processability with ∼90% of production yield, representing 52.5% enhanced electrical conductivity and 246% increased yield, respectively, compared to our in‐house MILD‐synthesized MXene. Also, HC‐Ti_3_C_2_T*_x_* MXene shows relatively higher electrical conductivity with ∼15 000 S/cm and production yield with ∼83.6%, showing 205% and 221% increase over our internal MILD control. The production yield and electrical conductivity values in Figure 5a were obtained from 5 to 7 independent experimental batches for each condition, and the error bars represent the corresponding standard deviations. Figure 5b provides a broader comparison of production yield and electrical conductivity from previous studies, distinguishing between N‐Ti_3_C_2_T*_x_* MXene and HC‐Ti_3_C_2_T*_x_* MXene. The SPEED method achieved superior results in both metrics, demonstrating its effectiveness in enhancing MXene synthesis efficiency and quality (Table S3). Additionally, various MXenes were synthesized by SPEED method as shown in Figure S11. Then, we compared the SPEED method with the MILD and mixed‐acid methods to find the reason why MXenes produced by SPEED method show higher electrical conductivity, which are established synthesis approaches. MXene synthesized by the SPEED method exhibited larger flake sizes, likely resulting in reduced inter‐flake resistance (Figure S12). Furthermore, TEM images show that this method produced high quality MXene with relatively low internal defects and minimal edge oxidation immediately after synthesis (Figure 5c–f), leading to lower intra‐flake resistance and improved electrical conductivity. AFM, SEM, and TEM analyses (Figure S13) further confirmed the uniform thickness (∼1.7 nm) and well‐defined morphology with low defect level of MXene synthesized by the SPEED method. TEM analysis (Figure 5e,f) showed no immediate differences in oxidation between SPEED and MILD‐synthesized HC‐Ti_3_C_2_T*_x_* MXene. However, after 10 days, hole defects formed on the basal plane in MILD sample, developing into larger pores that accelerated further oxidation (Figure S14). In contrast, the SPEED method resulted in fewer defects and less TiO_2_ formation. This improved oxidation stability is primarily attributed to the reduced defect density and Cl‐associated chemical stabilization of nascent Ti sites during the etching process. Previous reports showed that Cl can form thermally stable surface terminations on Ti_n+1_C_n_ frameworks, supporting the plausibility of Cl‐stabilized surface chemistry [41].

The oxidation stability of MXene synthesized by the SPEED and MILD methods was compared under accelerated aging conditions at 70°C to evaluate their relative oxidation resistance within a practical experimental time scale (Figure 5g; Figures S15 and S16) [4, 54]. Colloidal solutions of SPEED‐synthesized MXene exhibited delayed degradation, maintaining their dark color longer than MILD sample. Time constant (*τ*) analysis via UV–vis spectroscopy provided quantitative evidence of this stability and supports that MXenes synthesized by SPEED had significantly higher oxidation resistance than MILD synthesized samples (Figure 5h; Figure S17). Detailed equations and explanations are provided in the Supporting Information. The higher τ values of SPEED method indicate slower degradation kinetics than MILD method for both N‐MXene and HC‐MXene. In particular, N‐Ti_3_C_2_T*_x_* MXene synthesized by the MILD method displayed a rapid decline in intensity due to edge oxidation (Figure 5d). Additionally, MXene solutions synthesized via the SPEED method demonstrated superior stability under refrigerated conditions, with stable UV–vis peak at 785 nm and no TiO_2_ present for 90 to 120 days (Figure S18). Notably, additional XPS analysis after 9‐month refrigeration confirmed superior stability: SPEED‐MXene maintained low TiO_2_ content (∼11%) compared to MILD‐MXene (∼48%) (Figure S19). This proves that Cl‐passivation effectively preserves MXene`s metallic nature by inhibiting long‐term oxide nucleation.

### 3.2 Scalable and Durable Joule Heating Devices Utilizing Transparent Thin Film MXene

Furthermore, MXene synthesized using this method demonstrated superior performance in transparent conductive films and Joule heating devices, showcasing their potential for next‐generation electronic and energy systems (Figure 6; Figure S20). Transparent thin films of MXene were fabricated using a simple spin‐coating method, achieving uniform films across various sizes, from small dimensions (1 cm × 2 cm) to larger areas (10 cm × 10 cm), as shown in Figure 6a. HC‐MXene synthesized via the SPEED method demonstrated superior performance in terms of transmittance‐to‐sheet resistance ratio compared to those prepared using conventional methods. Generally, an increase in the MXene solution concentration resulted in a decrease in both transmittance and sheet resistance. Additionally, by adjusting the spin‐coating speed, variations in transmittance and sheet resistance could be achieved even at the same concentration. The heating performance of the MXene transparent thin film was evaluated by measuring the temperature changes over time. When a voltage of 12 V was applied, the average temperature increased progressively as the sheet resistance decreased (Figure 6b). This heat generation followed Joule's law, $H=\frac{V^{2}\;t}{R}\;$, demonstrating an inverse proportionality to the sheet resistance (*V* is the input voltage, *t* is time, and *R* is sheet resistance). The temperature of the MXene thin film increases proportionally with applied voltage, demonstrating effective voltage‐dependent heating (Figure 6c). The film exhibits rapid thermal response, reaching steady‐state temperature within seconds, with stable heating and cooling cycles. Furthermore, the film maintains stable operation even at higher voltages, highlighting its practicability for thermal applications. The MXene thin film was subjected to repetitive on/off cycles (60 s on, 60 s off), effectively oscillating between 35°C and 95°C. Importantly, the heating profile maintained its consistency from the initial to the final cycles, showcasing exceptional durability (Figure 6d). To the best of our knowledge, this represents among the highest operational cycles (330 cycles) reported for joule heating devices with MXene, underscoring the robust stability of the MXene thin film. The surface morphology was examined via SEM before and after 330 operational cycles, revealing no noticeable changes (Figure S21). To evaluate the stability under continuous operation, the device was driven at 10 V for 2 h. The temperature was stably maintained around 95°C throughout the operation without any interruptions, confirming its reliable performance under sustained heating conditions (Figure 6e).

**FIGURE 6:**
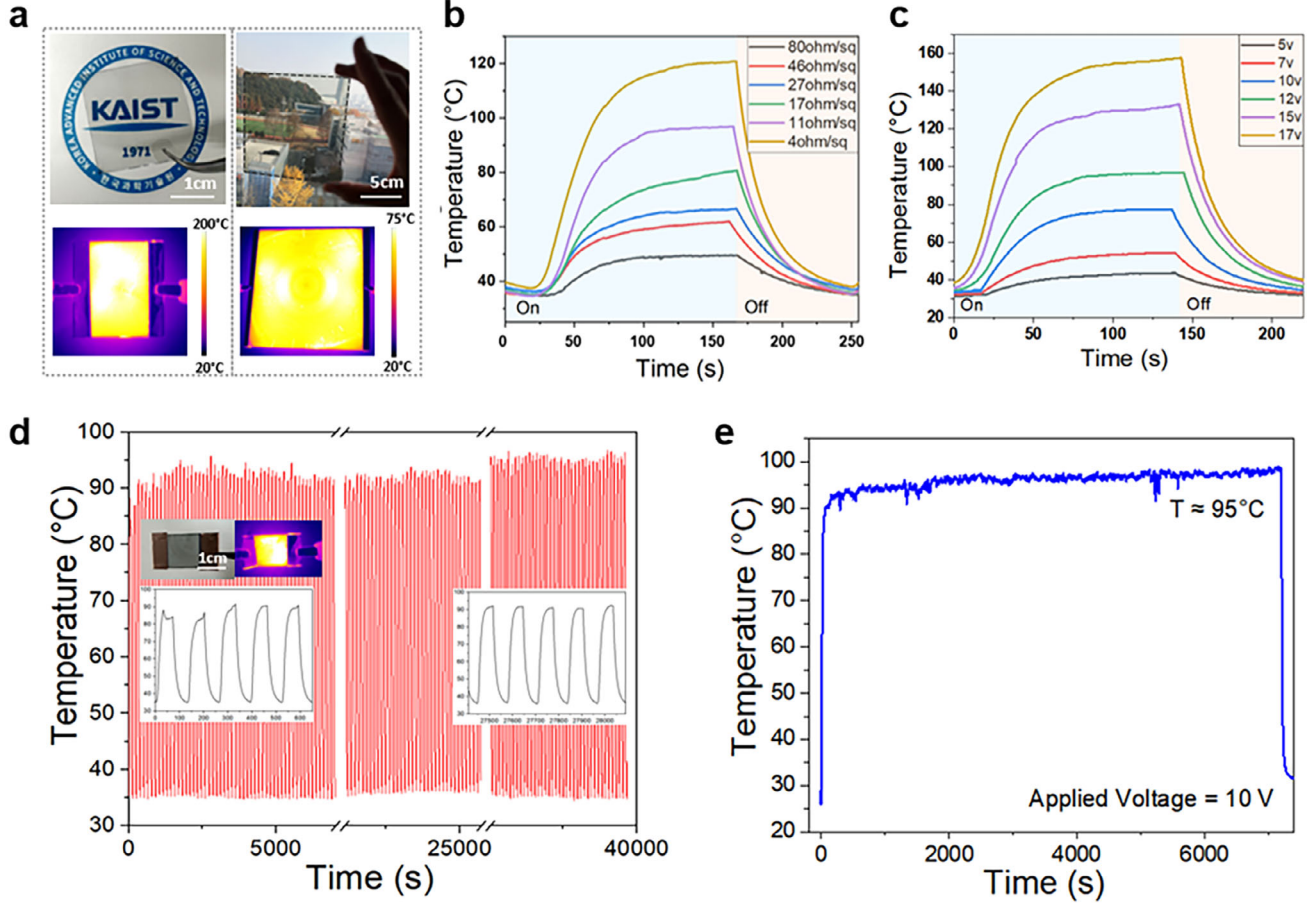
Heating performance and stability of transparent MXene thin films. (a) Photographs and IR images of MXene thin films with varying dimensions during heating. (b) Temperature profiles of films with different sheet resistances under constant voltage. (c) Voltage‐dependent temperature changes of MXene thin films. (d) Repetitive heating‐cooling cycles demonstrating durability. (e) Continuous heating stability under constant voltage operation.

## 4 Conclusion

In this study, we revealed a comprehensive understanding of the MXene etching and delamination process, uncovering the critical role of Cl^−^ ions in expanding interlayer spacing and facilitating maximized spontaneous delamination. Our findings highlight that the presence of both Cl^−^ and Li^+^ ions is essential from the etching process for achieving spontaneous delamination. Additionally, while Cl^−^ ions play a crucial role, the direct use of a small amount of HF is necessary for efficient grain boundary etching, which enhances the overall etching efficiency despite its potential to introduce minor defects. By combining systematic optimization of etching solutions with experimental observations and DFT calculations, we elucidated the chemical and structural dynamics underlying MXene synthesis. This systematic experimental approach enabled the development of the SPEED method (HF/HCl/LiCl), which achieved a synthesis yield of 83.6% and an electrical conductivity of ∼15 000 S/cm—substantially surpassing the performance of conventional methods such as MILD (LiF/HCl) and Mixed techniques (HF/HCl and LiCl).

The SPEED method not only enables the scalable production of high‐quality MXene with minimal defects, and uniform morphology across diverse MAX phases but also improves oxidation stability, significantly delaying material degradation under accelerated conditions. By advancing our understanding of the chemical and structural dynamics of MXene synthesis, this study addresses longstanding challenges in the field. These findings establish a robust framework for scalable MXene production and broaden their applicability across a wide range of high‐performance technologies.

## 5 Experimental Section/Methods

### 5.1 Synthesis of MXene

For the SPEED method, 0.5 g of N‐MAX, HC‐MAX, Mo_2_TiAlC_2_, Ti_3_AlCN, and Ti_2_AlC was used, while 0.25 g of V_2_AlC was used. The etchant was prepared by adding 7 mL of 50% HCl solution to 3 mL of deionized water, followed by 4 mL of 48% HF solution, and continuously stirring the mixture for 5 min. Subsequently, MAX powder was gradually added to the etchant over the course of 5 min, followed by LiCl with same weight of MAX powder. After etching for the specified time at room temperature, the reactant was washed by centrifugation with deionized water (5 min per cycle at 3500 rpm). When the pH reached 5–6, the solution was gently shaken to ensure thorough mixing of delaminated MXene supernatant and the precipitate that had not undergone delamination with unetched MAX. After completing the washing, 40 mL of deionized water was added to a 50 mL conical tube, and the tube was vigorously hand‐shaken for approximately 15 min until the concentration and viscosity of the solution increased. After that, the tube was placed on a shaker and shaken for an additional 15 min. The supernatant and precipitate were then separated by centrifugation (5 min at 3500 rpm), and only the supernatant was collected for further use. The remaining precipitate was then mixed with an additional 40 mL of deionized water and shaken on the shaker for 15 min. A second centrifugation (5 min at 3500 rpm) was performed to separate the second supernatant.

For MILD method, 0.8 g of LiF was added to 10 mL of 9 M HCl and left under continuous stirring for 5 min. For Mixed method, 20 mL of etchant was prepared by 6:3:1 mixture (by volume) of 12 M HCl, deionized water, 50 wt% HF. The process of adding MAX powder, washing, and delamination by shaking is the same as the one described above.

### 5.2 Calculation of Conversion Yield

The yield of the conversion of Ti_3_AlC_2_ into monolayer and few‐layer MXene was calculated using below calculation [55].

$$\begin{array}{ccc} & & \mathrm{Yield}\left(\%\right)\\ & & =\frac{\mathrm{Concentration}\;\mathrm{of}\;\mathrm{Supernatant}\left(\frac{\mathrm{mg}}{\mathrm{mL}}\right)\times \mathrm{Volume}\;\mathrm{of}\;\mathrm{Supernatant}\;\left(\mathrm{mL}\right)}{\mathrm{Weight}\;\mathrm{of}\;Ti_{3}\mathrm{Al}C_{2}\left(\mathrm{mg}\right)}\times 100 \end{array}$$

The detailed centrifuge‐assisted separation procedure, representative photographs of each fraction, and gravimetric validation of the yield calculation are provided in Figure S22. To ensure the reliability of these metrics, both production yield and electrical conductivity were derived from 5 to 7 independent experimental batches for each condition, with error bars representing the standard deviation.

## Funding

This work was supported by the National Research Foundation of Korea (NRF) through grants NRF‐2021M3H4A1A03047335, 2021M3H4A1A03047333, NRF‐RS‐2024‐00452934, and RS‐2025‐02223651, and NRF‐2021R1A6A3A01088168; the National Research Council of Science & Technology (NST) through grant CRC22034‐000; and the Korea Institute for Advancement of Technology (KIAT) through grant P0028332; and the National NanoFab Center (NNFC) through the Semiconductor‐Secondary Battery Interfacing Platform Technology Development Project (grant 2710109872).

## Conflicts of Interest

The authors declare no conflicts of interest.

## Supporting information




**Supporting File**: adma74422‐sup‐0001‐SuppMat.docx.

## Data Availability

The data that support the findings of this study are available from the corresponding author upon reasonable request.
