# Supplementary material for: Chloride Ions: Essential Agents for Achieving Spontaneous Delamination of MXene
Source: Adv Mater. 2026 Aug 19;38(58):e74422. doi: 10.1002/adma.74422 (PMC13674612; doi:10.1002/adma.74422)
Supplement: Supplementary file 1 [file ADMA-38-e74422-s001.docx]

Supporting Information

Chloride Ions: Essential Agents for Achieving Spontaneous Delamination of MXene

Sukhyeun Jang^1,2,8^, Yong-Jae Kim ^1,2,8^, Incheol Jeong^3,8^, Minju Kim^2^, Jaewoong Lee^1^, Jihan Kim^1^, Ki-Min Roh^3^, Kang Taek Lee^4^, Seon Joon Kim^5,6^*, Yonghee Lee,^7^* Chi Won Ahn^2^* and Hee-Tae Jung^1^*

^1^Department of Chemical and Biomolecular Engineering, Korea Advanced Institute of Science and Technology (KAIST), 291, Daehak-ro, Yuseong-gu, Daejeon 34141, Republic of Korea

^2^National Nanofab Center, Korea Advanced Institute of Science and Technology (KAIST), 291, Daehak-ro, Yuseong-gu, Daejeon 34141, Republic of Korea

^3^Resources Utilization Research Center, Korea Institute of Geoscience and Mineral Resources, Daejeon, 34132, Republic of Korea

^4^Department of Mechanical Engineering, Korea Advanced Institute of Science and Technology, 291, Daehak-ro, Yuseong-gu, Daejeon 34141, Republic of Korea

^5^Convergence Research Center for Solutions to Electromagnetic Interference for Future-mobility and Extreme Materials Research Center, Korea Institute of Science and Technology, 5, Hwarang-ro 14-gil, Seongbuk-gu, Seoul 02792, Republic of Korea

^6^Nano & Information Technology, KIST School, University of Science and Technology, 5, Hwarang-ro 14-gil, Seongbuk-gu, Seoul 02792, Republic of Korea

^7^Department of Nano & Advanced Materials Science and Engineering, Kyungpook National University, Gyeongsangbuk-do, 37224, Republic of Korea

^8^These authors contributed equally: Sukhyeun Jang, Yong-Jae Kim, Incheol Jeong

*E-mail: seonjkim@kist.re.kr, yhlee@knu.ac.kr, cwahn@nnfc.re.kr, heetae@kaist.ac.kr

**Synthesis of MAX phase (Ti_3_AlC_2_)**

Ti_3_AlC_2_ was synthesized using two methods: the mixture of Ti, TiC, and Al powders in a 2:1:1 molar ratio will be referred to as N-MAX, and the mixture in a 2:1:1 mass ratio will be referred to as Al-MAX. First, N-MAX was synthesized using the method from our group`s previous research.^[1]^ Briefly, Ti, TiC, and Al powders were mixed in the specified ratios and ball-milled for 18 hours using zirconia balls. The well-mixed powders were pressed under pressure to form pellets of uniform size and weight and then sintered at high temperature in an Ar gas atmosphere. The compacted pellets were crushed using a mortar and pestle and then sieved through a 200-mesh sieve to obtain particles smaller than 36 μm.

Al-MAX was synthesized based on the previous literature.^[26]^ The overall procedure is similar to that of the N-MAX method, except for HCl washing treatment to remove intermetallic impurities. Additional Ti_2_AlC and Ti_3_AlCN were purchased from Carbon (Ukraine). Mo_2_TiAlC and V_2_AlC were purchased from Nanochemazone (Canada).

**Characterization**

A Lejie LJS 280-1 shaker was used for mechanical shaking after handshaking. Scanning electron mi roscopy (SEM) images were obtained using a HITACHI SU8600 field-emission scanning electron microscope at an accelerating voltage of 5 kV and a working distance of 4.5 mm. X-ray diffraction (XRD) patterns were obtained using a Rigaku SmartLab high-resolution XRD system with Cu Kα1 radiation (λ = 1.5406 Å). The scans were performed with a step size of 0.01° and a scan speed of 2° min⁻¹. The diffraction data were analyzed using PDXL software and the ICDD PDF database. For XRD and SEM measurements, the washed samples were redispersed in water and drop-cast onto Si wafers, followed by drying under ambient conditions. Transmission electron microscopy (TEM) and Scanning transmission electron microscopy (STEM) images were acquired using a Cs-corrected JEOL JEM-ARM200F operated at 200kV. Additional high-resolution TEM and STEM analyses were performed using a Titan cubed G2 60-300 microscope equipped with a double-Cs corrector and Spectra Ultra. Energy-dispersive X-ray spectroscopy (EDS) analysis was performed using a Bruker QUANTAX 400 EDS system mounted on the JEOL JEM-ARM200F.

Cross-section TEM samples were prepared using a Helios Nanolab (FEI) dual-beam focused ion beam/scanning electron microscopy (FIB/SEM) system. After etching and washing, particles were drop-cast on Si wafers, and particles larger than 10 μm were selected for cross-sectional analysis. To minimize ion-beam-induced damage, Pt was deposited on the selected particles before FIB milling, and the samples were cut perpendicular to the c-direction. X-ray photoelectron spectroscopy (XPS) analysis was conducted using a Thermo fisher Scientific K-Alpha+ system equipped with a microfocused monochromatic Al Kα X-ray source (1486.7 eV). The analaysis area was selected within the range of 30-400 µm, and the measurements were performed under ultra-high vacuum conditions. Atomic force microscopy (AFM) analysis was performed using a Park Systems NX10 in non-contact mode. For AFM sample preparation, diluted MXene dispersions were drop-cast onto Si wafers and dried before measurement. AFM height images were typically acquired over 6 x 6 µm^2^ or 15 x 15 µm^2^ scan areas with a scan rate of 0.5 Hz and servo gain of 1. The flake thickness was determined from line-profile analysis across individual MXene flakes. UV-vis absorption spectra were obtained using a Cary 100 UV-vis spectrophotometer in absorbance mode over the wavelength range of 200-800 nm. The dispersion and oxidation stability of MXene aqueous dispersions was evaluated by monitoring the absorbance intensity at 785nm. The thickness of free-standing MXene films was measured using a Helios Nanolab (FEI) dual-beam system. Sheet resistance was measured using a four-point probe station (AIT, CMT-100MP). Temperature changes during Joule heating tests were monitored using an infrared camera (FLIR E75).

**Table S1.** **Effect of etching conditions on spontaneous delamination of MXene derived from different MAX phases.**

| **MAX phase** | **Acid** | **Ionic salt** | **Li^+^ addition** | **Delamination** |
| --- | --- | --- | --- | --- |
| Ti_3_AlC_2_ | HF | - | LiCl | No delamination |
|  | HF | - | LiF | No delamination |
|  | HF | LiF | - | No delamination |
|  | HF/HCl | - | LiCl | Delamination |
|  | HF/HCl | - | LiF | Delamination |
|  | HF | LiCl | - | Spontaneous Delamination |
|  | HCl | LiF | - | Spontaneous Delamination |
|  | HF/HCl | LiCl | - | Spontaneous Delamination |
|  | HF/HCl | LiF | - | Spontaneous Delamination |
| Ti_3_AlCN | HF/HCl | LiCl | - | Spontaneous Delamination |
| Mo_2_TiAlC_2_ |  |  | - | Spontaneous Delamination |
| V_2_AlC |  |  | - | Spontaneous Delamination |
| Ti_2_AlC |  |  | - | Spontaneous Delamination |

**Figure S1. Comparative SEM images of MXene flakes prepared via different chemical etching routes.** Individual panels display the resulting morphology under (a) MILD (LiF/HCl) etching, (b) pure HF etching, (c) Mixed acid (HF/HCl) etching, and (d) HF/LiCl etching, demonstrating the distinct flake integrity and lateral size variations depending on the ion intercalation environment.

**Figure S2. Cross-sectional HR-TEM images showing interlayer expansion under different etching conditions.** Representative HR-TEM images of (a) the parent MAX phase, (b) pure HF-etched, (c) HF/HCl-etched, and (d) HF/HCl/LiCl-etched frameworks. The lattice fringes show a gradual increase in interlayer spacing depending on the etching chemistry, supporting the role of Cl^-^-associated surface modification and Li^+^-assisted swelling in expanding the MXene interlayers.

**Figure S3. Cross-sectional STEM-EDS elemental mapping of etched MAX particle.** (a) Cross-sectional STEM image(scale bar: 300 nm). (b) Overlaid multi-elemental mapping image. Individual EDS maps for (c) Cl, (d) Al, (e) F, and (f) Ti, showing the spatial distribution of each element within the layered framenwork.

**Computational details**

DFT calculations were performed utilizing Vienna *ab-initio* simulation package (VASP)^[24]^. The Perdew–Burke–Ernzerhof generalized gradient approximation (GGA-PBE) was selected for the exchange-correlation functionals. Projector-augmented-wave potentials were used with valence configurations of 3*s*^2^3*p*^6^3*d*^3^4*s*^1^, 3*s*^2^3*p*^1^, and 2*s*^2^2*p*^2^ for Ti, Al, and C, respectively. Plane waves with an energy cutoff of 520 eV and a 6 × 6 × 1 k-point mesh within the Monkhorst–Pack scheme^[25]^ were determined for the unit cell of MAX, a 12-atom cell, with a convergence criterion of 0.01 eV/atom. The threshold for convergence of electronic self-consistent iterations was set to 10^−6^ eV/cell. The cell parameters and atomic positions were relaxed until the remaining force reached 5 × 10^−3^ eV/Å. A 5 × 1 × 1 supercell slab was used for the calculations of etching processes with a bottom layer fixed and 20 Å of vacuum along *a*-axis (Figure S4). A 3 × 3 × 1 supercell was used for the investigation of the interplanar distances and for first-principles molecular dynamics simulations. First-principles molecular dynamics simulations with the NVT ensemble were performed for 10ps with a time step of 1 fs at 500K. The shape and volume of the cells were fixed during the simulations. The binding energy of Li^+^ in the interlayer was calculated as

$$\text{E}_{\text{binding}}\text{ }\text{=}{\text{ }\text{E}}_{\text{MAX}}\text{ }\text{+}\text{ }\text{E}_{\text{Li}}-\text{ }\text{E}_{\text{MAX/Li}}$$

where${\text{ }\text{E}}_{\text{MAX}}$, $\text{E}_{\text{Li}}$, and$\text{ }\text{E}_{\text{MAX/Li}}$ are total energies of halogen-terminated MAX, a single Li atom in the bulk, and the Li intercalated MAX.

**Figure S4. A 5 × 1 × 1 supercell slab used for the calculations of etching processes**.

**Figure S5. DFT-calculated distance between two parent MAX layers during chemical etching.** Structural configurations and corresponding slit widths are compared when (a) F-terminated boundaries (5.6 Å) and (b) Cl-bound intermediates (9.0 Å) are formed.

**Table S2. Binding energy of Li^+^ for F^−^ and Cl^−^-terminated interlayers.**

| Termination | F^−^ | Cl^−^ |
| --- | --- | --- |
| Binding Energy (eV) | 0.69 | 0.38 |

**Optimization of the SPEED method.**

To achieve high yield and quality MXene, we refined the SPEED Method by optimizing etchant concentration, etching duration, and the conditions for delamination. The concentration of HF was found to significantly influence the quality of the resulting Mxene.^[5]^ In our experiments, amount of HCl (Figure S6) and LiCl (Figure S7), and HCl HF concentration ranging from 5 to 20 wt% were tested with reaction times of 12 and 24 hours. Initial tests were performed on the conventional Ti_3_AlC_2_ MAX phase (N-MAX), followed by the highly crystalline MAX phase (HC-MAX) at 20 wt% HF concentration. We observed distinct trends for electrical conductivity and yield based on both MAX phase type and reaction time (Figure S8). For N-MAX, electrical conductivity increased up to 12 hours but declined after 24 hours at 20wt% HF due to defect formation from over-etching (Figure S8a). In contrast, HC-MAX maintained high electrical conductivity even at 24 hours, suggesting improved structural stability under prolonged etching. Yield increased consistently with longer reaction times across all concentrations, with HC-MAX achieving the highest yield at 24 hours (Figure S9). Beyond 48 hours, over-etching led to a sharp decrease in both yield and electrical conductivity due to oxide material or carbide-derived carbon^[27,28]^. We further noted that the HF/LiCl method resulted in both lower yield and electrical conductivity compared to the SPEED method under the same HF concentration and reaction time. This underscores the critical role of HCl in facilitating both efficient etching and delamination.

To optimize the reaction for both high yield and electrical conductivity, it is ideal to use a high HF concentration with a shorter etching time, particularly for N-MAX.

**Figure S6. XRD patterns of MXene synthesized with different HCl volumes in HF/HCl/LiCl etching solutions.** The shift of the (002) reflection shows that the HCl content influences the interlayer expansion during etching.

**Figure S7. Optimization of the SPEED method based on the weight of LiCl added.** Comparative plots illustrating the simultaneous evaluation of production yield (black) and electrical conductivity (red) for (a) HC-MXene and (b) N-MXene.

**Figure S8. Optimization of SPEED process parameters under varying HF concentrations and etching durations.** (a) Electrical conductivity and (b) production yield of the MXene, comparing different precursor types (N-MAX and HC-MAX).

**Figure S9. Optimization of the SPEED etching window as a function of time.** (a) Quantitative table summarizing the evolution of production yield and electrical conductivity across different reaction durations. (b) Corresponding SEM images of the collected sediments sampled at specific intervals (4h, 8h, 12h, and 24h), visually distinguishing the structural stages from initial small-size MAX etching to the efficient processing range and subsequent over-etching behavior.

**Figure S10. Morphological comparison of the synthesized parent MAX powders.** (a) N-MAX grains prepared under stoichiometric sintering, displaying jagged edges, localized structural fragmentation, and a broad particle size distribution due to thermal loss. (b) HC-MAX grains synthesized with a 1.1–1.2 molar excess of Al, exhibiting well-developed layered morphology, highly intact grain structures, and smooth basal planes with a uniform size distribution.

**Table S3. Electrical conductivity and yield of MXene prepared by different methods**

(Conductivity= 1/sheet resistivity, resistivity= resistance*thickness)

| MAX Type | Etchant | Synthesis approach | Electrical conductivity  (S/cm) | Yield  (%) | Size (μm) | Reference |
| --- | --- | --- | --- | --- | --- | --- |
| HC-MAX | HF + HCl + LiCl | SPEED | 14966 | 83.6 | 4.5 | This work |
|  | HCl + LiF | Power-Focused Delamination | 25000 | 61.2 | 4.9 | ^[2]^ |
|  | HCl + LiF | Freeze-Sonication Delamination | - | 74.2 | 0.6 | ^[3]^ |
|  | HCl + LiF | Evaporated Nitrogen MILD | 24000 | 60 | 1.8 | ^[4]^ |
|  | HF + HCl / LiCl | Etching – Intercalation | >20000 | 30-50 | 1.1 | ^[5]^ |
|  | HF + HCl + LiF | MILD + HF | 18000 | 40 | 3 | ^[6]^ |
| N-MAX | HF + HCl + LiCl | SPEED | 7500 | 90 | 5.4 | This work |
|  | HF + HCl | Freeze-and-Thaw Assisted, Sonication | 9771 | 81 | 5.5 | ^[7]^ |
|  | HF + HCl | Soft Delamination | 6500 | 47 | 14 | ^[8]^ |
|  | HF | Hydrothermal Assisted Intercalation | 405 | 74 | 1 | ^[9]^ |
|  | HF | Microwave exfoliation | - | 10 | 2 | ^[10]^ |
|  | NaHF_2_ | Sonication | 2300 | 85 | 4 | ^[11]^ |
|  | HCl + LiF | Hand shaking & Sonication | 3669 | 80 | 1.5 | ^[12]^ |
|  | I_2_ | Iodine etching | 1250 | 71 | 1.8 | ^[13]^ |
|  | Electrochemical | Sonication | 1330 | 90 | 18.6 | ^[14]^ |
|  | NH_4_HF_2_ | Anhydrous etching | 8200 | 70 | 4.4 | ^[15]^ |
|  | HF + HCl | High pressure homogenization | 1500 | 80 | 0.8 | ^[16]^ |

**Versatility of the SPEED method for synthesizing various MXene**

Lastly, to verify if this synthesis method is applicable to other MAX phases, we conducted experiments with additional MAX phases: Ti_3_AlCN, Mo_2_TiAlC_2_, Ti_2_AlC and V_2_AlC. While many analysis methods are available, we quickly confirmed that delaminated MXene could be obtained from these phases through XRD and TEM analyses (Figure S11). Notably, the dispersion color varied slightly among the MXene phases, reflecting differences in synthesis and material characteristics. MXene with three M layers, such as Ti_3_CNT*_x_* and Mo_2_TiC_2_T*_x_*, demonstrated efficient etching and spontaneous delamination during the washing process. However, for MXene with two M layers, such as V_2_CT*_x_* and Ti_2_CT*_x_*, etching and delamination were less effective due to the lack of optimized reaction conditions, such as etching time and temperature. These samples did not exhibit spontaneous delamination, resulting in less effective exfoliation.

The shift of the (002) peak, indicative of increased interlayer spacing, along with TEM images and corresponding EDS mapping showing exfoliated single-layer MXene, confirms the capability of the SPEED method to synthesize MXene from various MAX phases. This versatility suggests the potential for broader application of the method, paving the way for synthesizing novel MXene with tailored properties.

**Figure S11. Generality of the SPEED method for synthesizing MXene from various MAX phase precursors.** Photographs of the resulting MXene dispersions, XRD patterns, and representative STEM-EDS analyses of MXene synthesized from (a) V₂AlC, (b) Ti₂AlC, (c) Ti₃AlCN, and (d) Mo₂TiAlC₂ using the SPEED method. The shift and broadening of the low-angle (002) reflections after etching indicate the formation of expanded layered MXene structures. Representative STEM-EDS maps show the expected elemental distributions in the resulting MXene flakes. These results demonstrate the feasibility of extending the SPEED chemistry to multiple MAX phase precursors, although further optimization may be required for each MXene composition.

**Figure S12. Lateral size distribution and representative SEM images of Ti₃C₂Tₓ MXene synthesized by different methods.** (a) Lateral size distributions of MXene flakes synthesized using the optimized SPEED condition, modified HF/HCl/LiCl conditions, the conventional MILD method, and the two-step etching/intercalation route. The average lateral sizes extracted from the fitted distributions are indicated in the plot. (b–e) Representative SEM images of MXene flakes prepared using (b) HF10/HCl/LiCl for 24 h, (c) HF20/HCl/LiCl for 12 h, (d) LiF/HCl for 24 h, and (e) HF10/HCl for 24 h followed by LiCl intercalation for 24 h.

**Figure S13. Morphological characterization of SPEED-synthesized Ti₃C₂Tₓ MXene.**
(a) AFM image of delaminated Ti₃C₂Tₓ MXene flakes on a substrate. (b) Representative height profile measured along the red line in (a), showing a flake thickness of approximately 1.7 nm. (c) Photographs of a freestanding MXene film demonstrating its macroscopic film-forming capability. (d) Photograph of the concentrated MXene dispersion after delamination. (e) SEM image of delaminated N-MXene flakes. (f) Low-magnification TEM image showing large-area MXene sheets. (g) High-resolution TEM image of the edge region marked by the red box in (f).

**Uv-vis spectroscopy analysis of degradation kinetics & long-term stability of MXene under refrigerated conditions**

To quantitatively assess these differences, Uv-vis spectroscopy was employed, offering a more precise evaluation of MXene stability. Absorbance spectra, along with spectra normalized to the local minimum at 220 nm, are presented in Figure S15 and S16. Here, the absorbance at 785 nm was selected as an indicator of MXene concentration^[29]^. By tracking the normalized intensity at this wavelength over time, we were able to systematically monitor the degradation kinetics of the colloidal solutions, providing insights into stability across different methods and conditions.

After 10 days in 70 ℃, oxidation appeared to accelerate, which can be attributed to the growth of pinhole defects on the basal plane. These defects gradually developed into larger pores, consequently leading to the formation of new edge-like domains. These newly formed edge sites likely served as active sites for further oxidation, thereby contributing to the rapid degradation of the material.

The time constant, τ, was calculated based on the equation A=A_unre_+A_re_e^-t/τ^, where A_unre_ and A_re_ denote the stable (unreactive) and reactive (unstable) MXene nanosheets, respectively.^[17][12]^ This analysis demonstrated that the SPEED method yields a significantly higher τ value, reflecting superior oxidation resistance in comparison to the MILD method (Figure S16).

However, Uv-vis and TEM analysis demonstrate that MXene synthesized via the SPEED method retains its properties effectively during refrigerated storage, underscoring its suitability for long-term use (Figure S18).

**Figure S14. TEM comparison of oxidized HC-Ti₃C₂Tₓ MXene after accelerated aging.** TEM images of HC-Ti₃C₂Tₓ MXene synthesized by (a) SPEED and (b) in-house MILD methods after aging at 70 °C for 10 days. SPEED-MXene shows fewer oxidation-induced defects, whereas the MILD control exhibits severe degradation with TiO₂ nanoparticle formation.

**Figure S15. Accelerated oxidation stability of N-Ti₃C₂Tₓ MXene dispersions monitored by UV–vis spectroscopy.** UV–vis absorbance spectra of N-Ti₃C₂Tₓ MXene dispersions synthesized by (a) the in-house MILD control and (b) the SPEED method during storage at 70 °C for up to 9 days. (c,d) Corresponding spectra normalized at 220 nm for (c) MILD- and (d) SPEED-synthesized MXene dispersions. (e) Photographs showing the time-dependent color change of SPEED- and MILD-synthesized MXene dispersions during accelerated aging at 70 °C.

**Figure S16. Accelerated oxidation stability of HC-Ti₃C₂Tₓ MXene dispersions monitored by UV–vis spectroscopy. UV–vis absorbance spectra of HC-Ti₃C₂Tₓ MXene dispersions synthesized by** (a) the in-house MILD control and (b) the SPEED method during storage at 70 °C for up to 16 days. (c,d) Corresponding spectra normalized at 220 nm for (c) MILD- and (d) SPEED-synthesized MXene dispersions. (e) Photographs showing the time-dependent color change of SPEED- and MILD-synthesized HC-Ti₃C₂Tₓ MXene dispersions during accelerated aging at 70 °C.

**Figure S17. Time constant analysis of MXene oxidation resistance.**

Time constants (τ) obtained by fitting the normalized UV–vis absorbance decay of N-MXene and HC-MXene dispersions synthesized by the in-house MILD control and SPEED methods. The higher τ values of SPEED-synthesized MXenes indicate slower degradation kinetics and enhanced oxidation resistance compared with the corresponding MILD controls.

**Figure S18. Long-term stability of SPEED-synthesized Ti₃C₂Tₓ MXene under refrigerated storage.**
TEM images after (a) 90 days and (b) 120 days of refrigerated storage. (c) UV–vis spectra of the MXene dispersion at 0, 90, and 120 days, indicating stable colloidal and optical characteristics during long-term storage.

**Figure S19. XPS of SPEED- and MILD-synthesized MXene after long-term refrigerated storage.** XPS survey spectra and high-resolution Ti 2p, O 1s, and C 1s spectra of (a) SPEED-MXene and (b) in-house MILD-control MXene after 9 months of refrigerated storage. SPEED-MXene shows a lower TiO₂-related component than the MILD control, indicating suppressed long-term oxidation.

**Figure S20. Optoelectronic properties of SPEED-synthesized MXene transparent films.**
(a) Transmittance and sheet resistance of MXene films prepared by spin coating at different MXene concentrations and spin-coating speeds. (b) Comparison of transmittance and sheet resistance of MXene films prepared in this work with previously reported MXene transparent conductive films. The SPEED-derived MXene films exhibit a favorable balance between optical transparency and electrical sheet resistance.

**Figure S21. Morphological stability of MXene transparent films after repeated Joule heating cycles.**
SEM images of the SPEED-derived MXene transparent film (a) before cycling and (b) after 330 heating/cooling cycles. The film surface retains a continuous and uniform morphology after repeated operation, indicating the structural durability of the MXene film under cyclic Joule heating conditions.

**Figure S22. Visual validation of high-yield delaminated MXene recovery during the SPEED process.**
(a) Photograph of the etched MXene slurry before washing. (b) Sequential photographs of the washing process, showing gradual neutralization and swelling of the etched MXene sediment. (c) Photographs of the concentrated MXene dispersion obtained after shaking. (d) Centrifuge-assisted separation of the exfoliation products, showing residual solid fractions containing multilayer MXene, partially etched MXene/MAX, and unetched MAX. (e) Photograph showing the small amount of residual sediment after supernatant collection. (f) SEM images of the residual sediment, showing remaining multilayer and MAX/MXene-containing particles.

To further validate the yield calculation, a defined volume of the collected MXene supernatant was vacuum-filtered to obtain a freestanding MXene film. The film was dried in a desiccator for more than 24 h to remove residual water and then weighed using a microbalance. The concentration of the supernatant was calculated from the dried film mass divided by the filtered volume. The total mass of few-layer MXene was then obtained by multiplying the concentration by the total supernatant volume.

During the centrifuge-assisted separation process, the exfoliation products were visually classified into several fractions: few-layer MXene in the supernatant, bulk/multilayer MXene in the upper sediment, partially etched MXene/MAX-containing sediment, and residual unreacted MAX at the bottom. This procedure confirms that the high yield reported for the SPEED method originates from efficient conversion of the etched precursor into few-layer MXene colloids rather than from residual sediment or unreacted MAX.

**Transparent MXene thin film heater: fabrication and measurement**

The substrate was cleaned by sonication in acetone, ethanol, and deionized water for 10 minutes each, followed by O_2_ plasma treatment for 3 minutes prior to spin coating. Subsequently, MXene solution was dropped onto the substrate and spin-coated at a certain speed for 120 seconds, with a ramp-up period of 6 seconds. After MXene coating and vacuum drying for 24 hours, copper foil electrodes were attached, and a DC power supply was used to apply the desired voltage. The temperature was monitored in real-time using an infrared (IR) camera during the measurement process, and the average temperature was measured and plotted as a graph by designating specific heating regions. Thin films with varying transmittance were successfully fabricated by adjusting the MXene solution concentration and spin-coating speed (Figure S20).^[20]^

**Table S4. Additional Sheet resistance vs. Transmittance Data**

| method | Sheet resistance (ohm/sq) | Transmittance (%) | Reference |
| --- | --- | --- | --- |
| Spin-coating | 25; 42; 51; 74; 106; 109; 200; 370; 422 | 44; 54; 52; 62; 75; 69; 86; 91; 92 | ^[18]^ |
| Spray-coating | 625; 8160 | 51; 81 | ^[19]^ |
| Spin-coating | 2010  3850  11870  23660 | 72  83  90  94 | ^[20]^ |
| Spin-coating | 440 8900 \| | 77  87 | ^[21]^ |
| Dip-coating | 375  600  1870 | 86  88  92 | ^[22]^ |
| Dip-coating | 40; 4300 | 51; 94 | ^[23]^ |

**Difference between the SPEED and MILD methods**

Unlike the conventional MILD method, in which HF is generated in situ from LiF/HCl and Al etching occurs simultaneously with Li⁺ intercalation, the SPEED process employs a single HF/HCl/LiCl etching solution. In this environment, directly supplied HF promotes efficient Al extraction, including etching through grain-boundary or defect-accessible pathways, while Cl⁻-assisted interlayer modification and Li⁺-assisted osmotic swelling occur together during etching. This single-pot ion-coupled process distinguishes SPEED from LiF/HCl-based MILD protocols and two-step HF/HCl etching followed by LiCl-assisted delamination.

**References**

[1]	Y.-J. Kim, S. J. Kim, D. Seo, Y. Chae, M. Anayee, Y. Lee, Y. Gogotsi, C. W. Ahn, H.-T. Jung, *Chem. Mater.* **2021**, 33, 6346.

[2]	Q. Zhang, R. Fan, W. Cheng, P. Ji, J. Sheng, Q. Liao, H. Lai, X. Fu, C. Zhang, H. Li, *Adv Sci (Weinh)* **2022**, 9, e2202748.

[3]	H. Song, Y. Ma, Q. Yao, C. Liu, X. Li, X. Tao, *Chem. Eng. J.* **2024**, 491.

[4]	A. Shayesteh Zeraati, S. A. Mirkhani, P. Sun, M. Naguib, P. V. Braun, U. Sundararaj, *Nanoscale* **2021**, 13, 3572.

[5]	M. Downes, C. E. Shuck, B. McBride, J. Busa, Y. Gogotsi, *Nature Protocols* **2024**.

[6]	A. Iqbal, J. Kwon, T. Hassan, S. W. Park, W.-H. Lee, J.-M. Oh, J. Hong, J. Lee, S. M. Naqvi, U. Zafar, S. J. Kim, J. H. Park, M.-K. Kim, C. M. Koo, *Adv. Funct. Mater.* **2025**, 35, 2409346.

[7]	X. Huang, P. Wu, *Adv. Funct. Mater.* **2020**, 30.

[8]	M. Shekhirev, J. Busa, C. E. Shuck, A. Torres, S. Bagheri, A. Sinitskii, Y. Gogotsi, *ACS Nano* **2022**, 16, 13695.

[9]	F. Han, S. Luo, L. Xie, J. Zhu, W. Wei, X. Chen, F. Liu, W. Chen, J. Zhao, L. Dong, K. Yu, X. Zeng, F. Rao, L. Wang, Y. Huang, *ACS Appl Mater Interfaces* **2019**, 11, 8443.

[10]	W. Wu, J. Xu, X. Tang, P. Xie, X. Liu, J. Xu, H. Zhou, D. Zhang, T. Fan, *Chem. Mater.* **2018**, 30, 5932.

[11]	Y. Zhao, M. Zhang, H. Yan, Y. Feng, X. Zhang, R. Guo, *Journal of Materials Chemistry A* **2021**, 9, 9593.

[12]	K. Rajavel, X. Yu, P. Zhu, Y. Hu, R. Sun, C. Wong, *ACS Appl Mater Interfaces* **2020**, 12, 49737.

[13]	H. Shi, P. Zhang, Z. Liu, S. Park, M. R. Lohe, Y. Wu, A. Shaygan Nia, S. Yang, X. Feng, *Angew. Chem. Int. Ed. Engl.* **2021**, 60, 8689.

[14]	S. Yang, P. Zhang, F. Wang, A. G. Ricciardulli, M. R. Lohe, P. W. M. Blom, X. Feng, *Angew. Chem. Int. Ed. Engl.* **2018**, 57, 15491.

[15]	T. Oh, S. Lee, H. Kim, T. Y. Ko, S. J. Kim, C. M. Koo, *Small* **2022**, 18, e2203767.

[16]	A. Inman, K. Shevchuk, M. Anayee, W. Hammill, J. Lee, M. Saraf, C. E. Shuck, C. M. Armstrong, Y. He, T. Jin, M. Shekhirev, J. Capobianco, Y. Gogotsi, *Chem. Eng. J.* **2023**, 475.

[17]	D. Hanlon, C. Backes, E. Doherty, C. S. Cucinotta, N. C. Berner, C. Boland, K. Lee, A. Harvey, P. Lynch, Z. Gholamvand, S. Zhang, K. Wang, G. Moynihan, A. Pokle, Q. M. Ramasse, N. McEvoy, W. J. Blau, J. Wang, G. Abellan, F. Hauke, A. Hirsch, S. Sanvito, D. D. O’Regan, G. S. Duesberg, V. Nicolosi, J. N. Coleman, *Nature Communications* **2015**, 6, 8563.

[18]	C. J. Zhang, B. Anasori, A. Seral-Ascaso, S. H. Park, N. McEvoy, A. Shmeliov, G. S. Duesberg, J. N. Coleman, Y. Gogotsi, V. Nicolosi, *Adv. Mater.* **2017**, 29.

[19]	K. Hantanasirisakul, M. Q. Zhao, P. Urbankowski, J. Halim, B. Anasori, S. Kota, C. E. Ren, M. W. Barsoum, Y. Gogotsi, *Advanced Electronic Materials* **2016**, 2.

[20]	M. Ebrahimi, C.-T. Mei, *Ceram. Int.* **2020**, 46, 28114.

[21]	M. Mariano, O. Mashtalir, F. Q. Antonio, W. H. Ryu, B. Deng, F. Xia, Y. Gogotsi, A. D. Taylor, *Nanoscale* **2016**, 8, 16371.

[22]	P. Salles, D. Pinto, K. Hantanasirisakul, K. Maleski, C. E. Shuck, Y. Gogotsi, *Adv. Funct. Mater.* **2019**, 29, 1809223.

[23]	P. Salles, E. Quain, N. Kurra, A. Sarycheva, Y. Gogotsi, *Small* **2018**, 14, 1802864.

[24]	G. Kresse, J. Furthmüller, *Phys. Rev. B* **1996**, 54, 11169.

[25]	H. J. Monkhorst, J. D. Pack, *Phys. Rev. B* **1976**, 13, 5188.

[26]	T. S. Mathis, K. Maleski, A. Goad, A. Sarycheva, M. Anayee, A. C. Foucher, K. Hantanasirisakul, C. E. Shuck, E. A. Stach, Y. Gogotsi, *ACS Nano* **2021**, 15, 6420.

[27]	M. Alhabeb, K. Maleski, B. Anasori, P. Lelyukh, L. Clark, S. Sin, Y. Gogotsi, *Chem. Mater.* **2017**, 29, 7633.

[28]	W. Sun, S. A. Shah, Y. Chen, Z. Tan, H. Gao, T. Habib, M. Radovic, M. J. Green, *J. Mater. Chem. A* **2017**, 5, 21663.

[29]	C. J. Zhang, S. Pinilla, N. McEvoy, C. P. Cullen, B. Anasori, E. Long, S.-H. Park, A. Seral-Ascaso, A. Shmeliov, D. Krishnan, C. Morant, X. Liu, G. S. Duesberg, Y. Gogotsi, V. Nicolosi, *Chem. Mater.* **2017**, 29, 4848.
